# Acupuncture reduces the time from extubation to ‘ready for discharge’ from the post anaesthesia care unit: results from the randomised controlled AcuARP trial

**DOI:** 10.1038/s41598-018-33459-y

**Published:** 2018-10-24

**Authors:** J. Fleckenstein, P. Baeumler, C. Gurschler, T. Weissenbacher, T. Annecke, T. Geisenberger, D. Irnich

**Affiliations:** 1 Department of Anaesthesiology, Ludwig-Maximilians University (LMU), Marchioninistraße 15, D-81377 Munich, Germany; 2 Department of Traditional Chinese Medicine/Acupuncture, Institute of Complementary Medicine IKOM, University of Bern, Personalhaus 4, Inselspital, CH-3010 Bern, Switzerland; 3 Department of Obstetrics and Gynaecology, Ludwig-Maximillians-University (LMU) Hospital, Maistraße 11, D-80337 Munich, Germany; 4 Department of Anaesthesiology and Intensive Care, University Hospital of Cologne, Uniklinik Köln, D-50924 Cologne, Germany; 5 Department of Anaesthesiology, Ospidal Engiadina Bassa, Via da l’Ospidal 280, CH-7550 Scuol, Switzerland

## Abstract

Acupuncture may improve peri-operative care as it reduces post-operative symptoms, such as pain, nausea and vomiting, or sedation. This patient-assessor blinded, randomised trial in 75 women undergoing gynaecologic laparoscopy evaluated the effects of acupuncture combined with a standardised anaesthetic regimen (ACU) on post-anaesthetic recovery, when compared to acupressure (APU) or standard anaesthesia alone (CON). Main outcome measure was the time from extubation to ‘ready for discharge’ from recovery as assessed by validated questionnaires. The main outcome differed significantly between groups (p = 0.013). Median time to ready for discharge in the ACU group (30 (IQR: 24–41) min) was 16 minutes (35%) shorter than in the CON group (46 (36–64) min; p = 0.015) and tended to be shorter than in the APU group (43 (31–58) min; p = 0.08). Compared to CON (p = 0.029), median time to extubation was approximately 7 minutes shorter in both, the ACU and the APU group. No acupuncture or acupressure-related side-effects could be observed. A difference in time to recovery of 16 minutes compared to standard alone can be considered clinically relevant. Thus, results of this study encourage the application of acupuncture in gynaecological laparoscopy as it improves post-anaesthetic recovery.

## Introduction

Modern surgery management demands for an increasing operating room turnover and more ambulatory surgeries. In order to meet these challenges post anaesthetic recovery needs to be optimized. To enhance recovery, it is recommended to prevent or minimize the occurrence of pain, post-operative nausea and vomiting (PONV), paralytic ileus, fatigue and sleep disturbances^1,2^. Also, patients should arrive in the post anaesthesia care unit (PACU) the least sedated as possible in order to reduce the period at which they are vulnerable to side effects of anaesthesia^3^. Acupuncture seems a promising non-medical complementary treatment option to minimize important factors that impede post anaesthetic recovery^4^.

There is substantial evidence provided by systematic reviews for the effectiveness of acupuncture in reducing post-operative pain, cumulative opioid consumption, opioid related side effects as well as pre-operative anxiety^5–8^. It was also suggested to introduce acupuncture and related techniques for treatment and prophylaxis of post-operative nausea and vomiting in routine clinical practice in combination with, or as an alternative to, conventional antiemetics. This is confirmed by an up to date Cochrane review showing no difference between PC6 acupuncture point stimulation and antiemetic drugs to prevent PONV^9^. Accordingly the use of acupuncture is generally recommended in the consensus guidelines for the management of PONV by the Society for Ambulatory Anaesthesia^10^.

Based on this evidence we hypothesized that acupuncture may accelerate the time from extubation to ‘ready for discharge’ from the PACU, this being considered a meaningful indicator of post-anaesthetic recovery. In addition, this study investigated whether specific effects related to penetration of the skin would render press needle acupuncture superior to acupressure with non-penetrating press plasters^11^.

## Patients and Methods

### Study Design

This single-centre, patient-assessor blinded, randomised controlled study compared the effect of press needle acupuncture (ACU) and press plaster acupressure (APU) versus no treatment (CON) on post-operative recovery in a standard anaesthetic setting of programmed gynaecologic laparoscopic surgeries. Main outcome measure was the time from extubation to ‘ready for discharge’ from the PACU. Analysis of all records was performed by blinded assessors. The total follow-up period per patient was at least two days. The study took place at the Department of Gynaecology, Ludwig-Maximilians-University, Munich, Germany. The study was approved by the Ethics Committee of the University of Munich, Germany (reference 009–12) and is in agreement with the Declaration of Helsinki (Version Fortaleza 2012). Trial registration is NCT01816386. The protocol has previously been published^11^. The following paragraphs describe the study methods in brief.

### Patients

Female patients scheduled for laparoscopic surgery of the uterus, the adnexa or the ovaries with an ASA-score ≤ 2 were screened for this study. Patients were included after signing written informed consent. For inclusion and exclusion criteria please refer to Table 1.

**Table 1:** Inclusion and Exclusion Criteria.

Inclusion Criteria	General Exclusion Criteria	Indication-specific Exclusion Criteria
•Age 18+ •Females scheduled for laparoscopic surgery of uterus, adnexa or ovaries •American Society of Anaesthesiologists (ASA)-score ≤2 •Ability to follow study instructions and likely to attend and complete all required visits •Written informed consent	•Subject without legal capacity •Subject who is unable to understand the nature, scope, significance and consequences of this clinical trial •Simultaneously participation in another clinical trial or participation in any clinical trial involving administration of acupuncture within 30 days prior to inclusion •Subjects with a physical or psychiatric condition which at the investigator’s discretion may put the subject at risk, may confound the trial results, or may interfere with the subject’s participation in this clinical trial •Known or persistent abuse of medication, drugs or alcohol •Current or planned pregnancy or nursing women •Females of childbearing potential, who are not using and not willing to use medically reliable methods of contraception for the entire study duration	•Surgery within the last three months •Chronic pain > three months •Continuous analgesic medication with opioids longer than three day •Massive degenerative diseases •Pre-treatment with acupuncture or trigger point injection within the last two months

### Randomised treatment allocation, blinding and sample-size estimation

Patients were informed, both orally and in writing, that this study would distinguish two forms of interventions (APU and ACU)^12^, and about the possibility of 33% to be allocated to a control group, where they would receive the same anaesthetic care and attention by the study team as all other participants.

Following written informed consent, patients were randomly assigned to either ACU, CON or APU by using the internet based randomization software RANDOULETTE^®^ (Institute of Medical Information Sciences, Biometry and Epidemiology, Ludwig-Maximilians-University of Munich). Blinding of patients and assessors was ensured by using press needles & press plasters^12^. Patients in the control group could not be blinded.

The sample size of 75 patients was estimated on the basis of a supposed small to medium effect (Cohen’s d = 0.4) on post-operative recovery, an alpha-error of 5%, a power of 80% and a drop-out rate of 15% using G*Power (Version 3.1.3, University of Düsseldorf, Germany)^11^. The estimated time to recovery following gynaecologic laparoscopy in our clinic has been observed to range from 45 to 60 minutes, similar to published data resulting from mini cholecystectomy^13^. Shortening this time by 25% (i.e. 11 to 15 minutes) seems to be clinically meaningful^14^.

### Anaesthetic Proceedings

The anaesthetic procurements were standardised and based on general recommendations and guidelines of the German Society of Anaesthesiology (DGAI) and clinic standards, and have previously been described in depth^11^. Opioids and propofol were administered by Target Controlled Infusion (TCI; Fresenius-Kabi Orchestra® Base Primea syringe pumps, Fresenius Kabi Group, Bad Homburg, Germany). The target effect-site concentrations for the induction (maintenance) of anaesthesia were 0.2–0.4 (0.12–0.22) ng/ml sufentanyl and 3.0 to 9.0 (3.0–4.0) µg/ml propofol.

All patients received metamizol 2.5 g intra-venous (i.v.)-infusions during the last 30 minutes of the surgery to prevent post-operative pain, dexamethasone 8 mg i.v. (after induction) to prevent PONV and ranitidine 50 mg i.v. (after induction) for prevention of gastric stress ulcer.

Post-operative pain therapy included metamizol (4*1.25 g/day, 6 hours interval) on demand. In case of nausea and vomiting or shivering patients were treated according to the clinical standard.

### Interventions

At the time of the pre-anaesthetic visit, 12–24 hours prior to surgery, patients in the intervention groups received a standardised treatment with either 12 press needles (ACU; sharp tip; 0.2 mm × 1.5 mm) or 12 press plasters (APU; blunt knob; both Seirin New Pyonex^®^, Seirin Corp., Shizuoka City, Japan) at GV26, a point described to resolve states of sedation and collapse^15,16^ and 6 acupuncture points which have been proposed to relief pain and strengthen the constitution, i.e. CV17 (on the middle body line), and bilateral LI4, HT7, LR3, ST36 and PC6)^11^. Deqi response is usually not elicited when applying press needles, and was consequently not required. Needles or plasters were left in place for 72–96 hours depending on the patient’s discharge from the hospital (earliest 48 hours after surgery), covering the whole perioperative period. Treatments were performed by three acupuncturists with at least 120 hours acupuncture training. (A-Diploma standard of the German Medical Acupuncture Association DÄGfA).

Patients were instructed to press the needles or plasters as often as wanted, especially when suffering from anxiety, pain or nausea and vomiting. Three daily visits were established to guarantee at least three stimulations per day. During emergence, i.e. starting with the end of anaesthetic drugs, the trial team was required to stimulate at the acupuncture point GV26.

The control group underwent the same standard anaesthetic procedure, received the same amount of peri-operative visits, and the same empathy and motivation by the study team.

### Blinding procedure

In this study a patient and assessor blind situation regarding the use of press needles versus press plasters was achieved. Neither patients nor examiners were able to distinguish between these two interventions^12^, as both parties do not know if a sharp needle (acupuncture) or a blunt knob (acupressure) was located below the plaster^11^. However the patients in the control group could not be blinded, as they were aware of not receiving an additional treatment. Also assessors could not be blinded with regard to the control group, as patients did not carry plasters.

### Outcome measures

#### Main outcome measure

The main outcome measure, was the time from extubation of the trachea to ‘ready for discharge’ from the PACU as assessed by three independent recovery scores (Aldrete^17^, Post Anaesthetic Discharge Scoring System^18^, in-house score for outpatients; for details please refer to the study protocol)^11^. All scores consist of five items regarding different postoperative physiologic states of the patient, which are graded from 0 to 2. A summated score of 9 to 10 indicates that the patient is ready for discharge. When fulfilling all scores, the patient was graded as ready for discharge.

#### Secondary outcome measures

Secondary outcomes have been previously been described^11^. Acupuncture-related outcomes included pre-operative anxiety, depth of sedation (bispectral index; BIS), reaction times during emergence, time to extubation, post-operative analgesic consumption and pain intensity, and the occurrence of anaesthesia-related side effects.

#### Statistical analysis

Data entry was carried out twice. Analysis was performed with the SPSS statistical software system (SPSS Inc., Chicago, IL; version 23.0). Fisher test was used to compare binary outcomes between the control and the intervention groups. Metric-variables were assessed for normal-distribution by Kolmogorov-Smirnov-Test. For normally distributed variables, the mean and standard deviation (SD) were calculated for descriptive analyses, and groups were compared by an analysis of variance (ANOVA). The unpaired t-Test was used for post-hoc pairwise comparisons. Influence of group x time of the state anxiety was assessed by ANOVA for repeated measures with group as between subject factor. In case of a non-normal distribution, non-parametric analyses were performed with descriptive statistics given as median (M) and interquartile-range (IQR). Kruskal-Wallis-Test was used for intergroup comparisons. Dunn’s Test was used for post-hoc pairwise group comparisons.

In order to address the bias possibly induced by unequal surgery durations, a sensitivity analysis excluding cases with extremely short, extremely long or missing surgery times. Extreme values were identified from the graphical display of individual surgery times per group.

## Results

**Figure 1:**
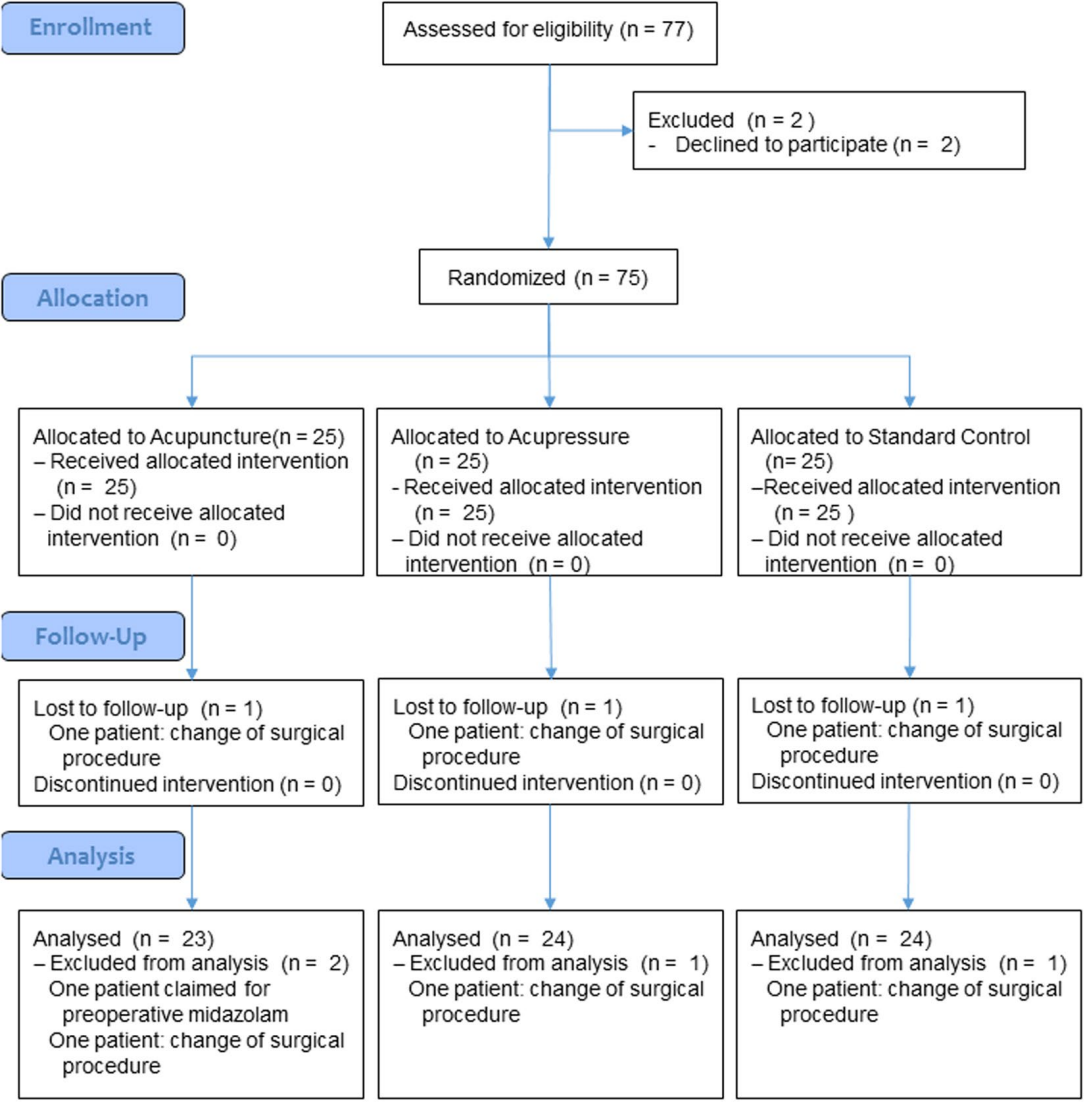
Consort study flow chart.

Seventy-five women (age (mean (SD)) 44 (12) years, height 167 (7) cm, weight (median (IQR)) 65 kg (58–78), pain intensity at admission 0.1 (0.0–0.3) cm on a 10 cm visual analogue scale, VAS) were included and randomised into the study. Four patients dropped out of the study; two due to a conversion of the surgery to an open procedure (n = 2), one patient claimed for midazolam instead of continuing in the study, and one patient received midazolam accidentally (Fig. 1). Surgical sites were adnexa 56%, uterus 37% or both 7%. Patient characteristics were similar in the three study groups with the exception of body weight that was highest in the ACU group (Table 2).

**Table 2:** Patients demographic characteristics.

		Total (n = 71)	APU (n = 24)	CON (n = 24)	ACU (n = 23)	p-value
Age (years), range		19–72	26–69	26–72	19–71	^a^0.765
Height (cm), mean (SD)		167.2 (7.0)	169.4 (7.1)	167.0 (6.9)	165.0 (6.7)	^a^0.102
Weight (kg), M (IQR)		65.0 (58.0–78.0)	71.5 (61.5–81.5)	62.5 (58.3–70.5)	63.5^#^ (55.0–72.0)	^b^ 0.031*
Civil status n (%)	Single	33 (46.5)	9 (37.5)	11 (45.8)	13 (56.5)	
	Married	28 (39.4)	11 (45.8)	11 (45.8)	6 (26.1)	
	Widowed	2 (2.8)	1 (4.2)	0 (0.00)	1 (4.3)	
	Divorced	8 (11.3)	3 (12.5)	2 (8.3)	3 (13.0)	
Religion n (%)	Protestant	8 (11.4)	2 (8.7)	1 (4.2)	5 (21.7)	
	Catholic	39 (55.7)	13 (56.5)	14 (58.3)	12 (52.2)	
	Islam	4 (5.7)	2 (8.7)	2 (8.3)	0 (0.0)	
	Others/none	19 (27.1)	6 (26.1)	7 (29.2)	6 (26.1)	
Education n (%)	Main school	6 (8.5)	4 (16. 7)	2 (8.3)	0 (0.00)	
	Secondary school	21 (29.6)	7 (29.1)	4 (16.7)	10 (43.5)	
	A-level	11 (15.5)	2 (8.3)	6 (25.0)	3 (13.0)	
	College degree	33 (46.5)	11 (45.8)	12 (50.0)	10 (43.5)	
Profession n (%)	Student	4 (5.7)	1 (4.2)	1 (4.2)	2 (9.1)	
	Blue collar worker	2 (2.9)	2 (8.3)	0 (0.00)	0 (0.0)	
	Employee	44 (62.9)	13 (54.2)	17 (70.8)	14 (63.6)	
	Public official	2 (2.9)	1 (4.2)	0 (0.0)	1 (4.5)	
	Retired	3 (4.3)	0 (0.0)	2 (8.3)	1 (4.5)	
	House wife	6 (8.6)	4 (16.7)	2 (8.3)	0 (0.0)	
	Unemployed	2 (2.9)	2 (8.3)	0 (0.0)	0 (0.0)	
	Self-employed	7 (10.0)	1 (4.2)	2 (8.3)	4 (18.2)	
Type of surgery	Uterus	26 (36.6)	11 (45.8)	7 (29.2)	8 (34.8)	
	Adnexa	40 (56.3)	13 (54.2)	14 (58.3)	13 (56.5)	
	Uterus & Andexa	5 (7.0)	0 (0.0)	3 (12.5)	2 (8.7)	
Pain at admission (VAS, 0–10 cm), M (IQR)		0.1 (0.0–0.3)	0.1 (0.0–0.29)	0.1 (0.0–0.65)	0.0 (0.0–0.2)	0.395

APU: press plaster acupressure, CON: control, ACU: press needle acupuncture, n: case number, SD: standard deviation, M: median, IQR: interquartilerange, ^a^ANOVA, ^b^Kruskal-Wallis-Test, *significant overall group difference on an alpha level of 5%, ^#^significantly different from the APU group on an alpha level of 5%, post-hoc *pairwise group* comparisons were performed by Dunn’s Test.

**Figure 2:**
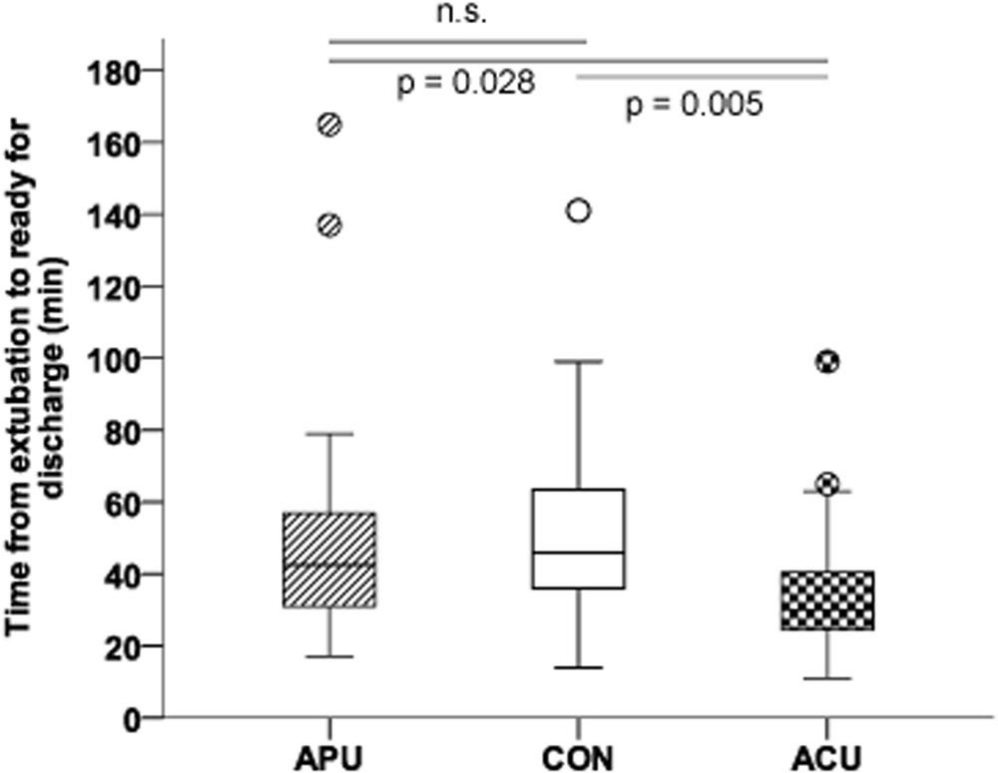
Times to recovery. The figure indicates the time from extubation to ready for discharge from the PACU, with acupuncture showing the fastest time to full recovery. The figure shows boxplots (with median and IQRs) as well as outliers (circles). Between groups analysis revealed significant differnces (Kruskal-Wallis p = 0.013). P-values are given for the post-hoc pairwise comparisons as performed by Dunn’s test. APU: press plaster acupressure, CON: control, ACU: press needle acupuncture.

The main outcome parameter, time from extubation to ‘ready for discharge’ from the PACU was significantly decreased in the ACU group (median (IQR) 30 (24–41) min; Kruskal-Wallis-Test p = 0.013) when compared to the CON group (46 (36–64) min; post-hoc Dunn’s Test p = 0.005) and the APU group (43 (31–58) min; post-hoc Dunn’s Test p = 0.028; see Table 3 and Fig. 2). The median time to ready for discharge from the PACU in the ACU group was 35% less than in the CON group, and 29% less than in the APU group to fulfil all the discharge criteria. There were no significant differences in the time to extubation as calculated by the TCI pumps (see Table 4).

**Table 3:** Anesthetic recovery period.

		Total (n = 71)	APU (n = 24)	CON (n = 24)	ACU (n = 23)	p-value
**Operating theater**						
Time TCI off to BIS 70 (min)	*n*	69	22	24	23	
	*M (IQR)*	10.0 (6.5–16.0)	10.0 (5.0–11.3)	12.5 (9.0–17.8)	8.0 (6.0–19.0)	0.069
Time TCIoff to extubation (min)	*n*	71	24	24	23	
	*M (IQR)*	16.0 (10.0–22.0)	13.0 (9.3–20.0)^#^	19.0 (14.0–25.8)	12.0 (7.0–22.0)^#^	0.029*
Time from induction of anesthesia to suture (min)	*n*	64	23	21	20	
	*M (IQR)*	107.0 (97.3–145.0)	112.0 (101.0–155.0)	100.0 (91.5–113.0)	128.5 (86.5–150.5)	0.114
**Main outcome**						
Time to ‘ready for discharge’ from PACU (min)	*n*	71	24	24	23	
	*M (IQR)*	40.0 (28.0–55.0)	42.5 (31.0–58.0)	46.0 (36.0–63.8)	30.0 (24.0–41.0)^#,§^	0.013*

APU: press plaster acupressure, CON: control, ACU: press needle acupuncture, n: case number, M: median, IQR: interquartilerange, min^−1^: per minute, mmHg: millimeters of mercury, BIS: bispectral index, min: minute(s), TCIoff: stop of target controlled infusion, O_2_: Oxygen, PACU: post-anaesthesia care unit, *significant on an alpha level of 5% according to Kruskal-Wallis-Test, ^#^significantly different from the CON group. ^§^Significantly different from APU group. Post-hoc pairwise group comparisons were performed by Dunn’s Test.

**Table 4:** Acupuncture-related secondary outcomes.

			Total (n = 71)	APU (n = 24)	CON (n = 24)	ACU (n = 23)	p-value
**Pre-operative anxiety**							
***Baseline***							
*STAI-State*	n		66	22	22	22	0.482
	mean (SD)		42.5 ± 9.0	42.2 ± 11.2	41.0 ± 8.0	44.3 ± 7.4	
*STAI-Trait*	n		68	22	23	23	0.075
	mean (SD)		37.0 ± 9.1	39.1 ± 9.0	33.5 ± 7.1	38.5 ± 10.2	
***Morning of surgery***							
*STAI-State*	n		65	19	23	23	0.788
	mean (SD)		45.0 ± 10.7	43.9 ± 12.7	46.2 ± 11.9	44.8 ± 7.6	
*STAI-Trait*	n		64	20	23	21	0.267
	mean (SD)		37.2 ± 9.3	39.5 ± 9.6	34.9 ± 7.7	37.4 ± 10.4	
***Prior to induction***							
*STAI-State*	n		71	24	24	23	0.196
	mean (SD)		48.3 ± 11.4	45.0 ± 12.2	50.8 ± 12.7	49.2 ± 8.5	
*STAI-Trait*	n		70	24	23	23	0.379
	mean (SD)		36.3 ± 9.9	37.1 ± 9.6	33.9 ± 7.5	37.7 ± 12.1	
**Induction of anaesthesia**							
*BIS at induction*	*n*		71	24	24	23	0.029*
	*M (IQR)*		98.0 (97.0–98.0)	98.0 (97.0–98.0)	98.0 (98.0–98.0)	97.0 (96.0–98.0)^#^	
*BIS at 5 minutes after induction*	*n*		71	24	24	23	0.78
	*M (IQR)*		43.0 (36.0–56.0)	44.5 (34.0–58.3)	47.0 (35.5–56.0)	41.0 (37.0–49.0)	
*Sufentanyl at intubation (µg)*	*n*		68	23	23	22	0.54
	*M (IQR)*		26.3 (21.1–33.5)	24.2 (20.5–30.8)	26.8 (21.1–34.0)	28.0 (21.2–34.3)	
*Propofol at intubation (mg)*	*n*		68	23	23	22	0.891
	*M (IQR)*		261.0 (198.0–299.3)	263.0 (214.0–294.0)	260.0 (218.0–293.0)	243.5 (184.8–327.8)	
*Mivacurium at intubation (mg)*	*n*		55	21	17	17	0.533
	*M (IQR)*		13.8 (12.0–16.0)	14.0 (12.1–16.0)	13.0 (12.0–14.8)	12.8 (11.5–17.7)	
*Atracurium at intubation (mg)*	*n*		16	3	7	6	0.805
	*M (IQR)*		30.0 (30.0–30.0)	30.0 (30.0–30.0)	30.0 (30.0–30.0)	30.0 (27.5–42.5)	
*Effect Site Conc. sufentanyl*_*BIS50*_	*n*		71	24	24	23	0.414
	*M (IQR)*		0.4 (0.3–0.5)	0.4 (0.3–0.5)	0.4 (0.3–0.5)	0.3 (0.3–0.5)	
*Effect Site Conc. propofol*_*BIS50*_	*n*		70	24	23	23	0.925
	*M (IQR)*		7.0 (5.9–7.9)	7.0 (6.0–7.5)	7.0 (5.8–8.0)	6.7 (5.8–7.8)	
*Time from induction to cut (min)*	*n*		70	23	24	23	0.799
	*M (IQR)*		42.0 (36.0–50.0)	39.0 (36.0–53.0)	45.5 (37.0–54.0)	42.0 (36.0–48.0)	
**End of anaesthesia**							
*BIS at TCIoff*	*n*		71	24	24	23	0.020*
	*M (IQR)*		37.0 (27.0–44.0)	41.5 (27.3–49.8)^#^	31.5 (24.0–38.8)	38.0 (31.0–50.0)^#^	
*BIS at first breathing*	*n*		71	24	24	23	0.664
	*M (IQR)*		70.0 (57.0–77.0)	70.0 (55.5–76.0)	72.5 (54.3–83.0)	68.0 (57.0–74.0)	
*BIS at extuabtion*	*n*		71	24	24	23	0.222
	*M (IQR)*		80.0 (75.0–84.0)	81.5 (76.3–83.8)	83.0 (75.5–87.3)	76.0 (74.0–82.0)	
*Effect site conc. Sufentanyl*_*TCIoff*_ *(ng/ml)*	*n*		71	24	24	23	0.072
	*M (IQR)*		0.1 (0.1–0.2)	0.1 (0.1–0.1)	0.1 (0.1–0.2)	0.1 (0.1–0.2)	
*Effect site conc.Propofol*_*TCIoff*_ *(µg/ml)*	*n*		71	24	24	23	0.951
	*M (IQR)*		2.8 (2.2–3.5)	2.7 (2.1–3.5)	2.9 (2.1–3.5)	2.6 (2.3–3.5)	
*Total sufentanyl (µg)*	*n*		71	24	24	23	0.615
	*M (IQR)*		43.8 (39.8–48.7)	46.5 (40.0–52.9)	44.3 (39.1–46.3)	42.5 (38.8–47.7)	
*Total propofol (mg)*	*n*		69	22	24	23	0.079
	*M (IQR)*		1197.0 (975.0–1594.0)	1300.0 (1092.8–1793.0)	1109.0 (930.5–1333.5)	1251.0 (932.0–1598.0)	
*Total mivacurium (mg)*	*n*		55	21	17	17	0.233
	*M (IQR)*		30.0 (26.0–36.0)	29.0 (25.0–35.0)	26.6 (24.5–33.0)	30.5 (27.0–45.0)	
*Total atracurium (mg)*	*n*		16	3	7	6	0.805
	*M (IQR)*		30.0 (30.0–30.0)	30.0 (30.0–30.0)	30.0 (30.0–30.0)	30.0 (27.5–42.5)	
*Time to awakening sufentanyl*_*TCIoff*_ *(min)*	*n*		71	24	24	23	0.098
	*M (IQR)*		11.0 (1.0–22.0)	7.0 (0.0–18.8)	16.0 (5.5–29.0)	11.0 (0.0–23.0)	
*Time to awakening propofol*_*TCIoff*_ *(min)*	*n*		71	24	24	23	0.281
	*M (IQR)*		9.0 (6.0–15.0)	8.0 (6.3–13.0)	8.0 (6.0–16.8)	12.0 (6.0–18.0)	
*Effect site conc. sufentanyl*_*Extubation*_ *(ng/ml)*	*n*		71	24	24	23	0.997
	*M (IQR)*		0.1 (0.1–0.1)	0.1 (0.1–0.1)	0.1 (0.1–0.1)	0.1 (0.1–0.1)	
*Effect Site Conc. propofol*_*Extubation*_ *(µg/ml)*	*n*		70	24	24	22	0.136
	*M (IQR)*		1.1 (0.9–1.3)	1.0 (0.9–1.3)	1.0 (0.8–1.1)	1.1 (1.0–1.4)	
*Time from induction to TCIoff (min)*	*n*		71	24	24	23	0.048*
	*M (IQR)*		100.0 (84.0–138.0)	105.0 (95.0–158.5)^#^	91.5 (84.0–103.3)	123.0 (75.0–143.0)	
*Duration of surgery (min)*	*n*		63	22	21	20	0.035*
	*M (IQR)*		65.0 (49.0–95.0)	72.5 (55.0–114.5)^#^	56.0 (44.0–70.5)	84.5 (52.0–106.8)^#^	
**Pain (VAS, 0–10 cm)**							
*End of PACU*	n		68	24	23	21	0.87
	*M (IQR)*		1.0 (0.0–2.0)	1.0 (0.0–2.0)	1.0 (0.0–2.0)	1.0 (1.0–2.0)	
6 h post-surgery	n		70	23	24	23	0.365
	*M (IQR)*		2.1 (0.7–3.1)	2.3 (0.6–4.1)	2.2 (0.8–4.1)	1.7 (0.5–2.7)	
*d1 8°°*	n		71	24	24	23	0.335
	*M (IQR)*		1.3 (0.6–2.7)	1.1 (0.3–3.1)	1.9 (0.9–3.1)	1.2 (0.5–2.1)	
*d1 14°°*	n		68	24	21	23	0.712
	*M (IQR)*		1.1 (0.4–2.7)	0.7 (0.1–3.2)	1.7 (0.7–2.9)	1.3 (0.5–1.9)	
*d1 20°°*	n		69	24	22	23	0.346
	*M (IQR)*		1.0 (0.3–2.2)	0.7 (0.1–2.0)	1.3 (0.4–2.3)	1.1 (0.5–1.9)	
*d2 8°°*	n		68	24	21	23	0.697
	*M (IQR)*		0.5 (0.1–1.7)	0.3 (0.0–2.6)	1.0 (0.2–1.8)	0.6 (0.2–1.2)	
*d2 14°°*	n		60	22	18	20	0.737
	*M (IQR)*		0.6 (0.2–1.6)	0.7 (0.0–1.5)	0.5 (0.2–2.6)	0.6 (0.3–1.1)	
*d2 20°°*	n		53	20	17	16	0.592
	*M (IQR)*		0.3 (0.1–1.2)	0.3 (0.0–1.3)	0.3 (0.1–1.4)	0.5 (0.2–1.1)	
	Total (n = 71)		APU (n = 24)	p-value ^vs CON^	CON (n = 24)	ACU (n = 23)	p-value ^vs CON^
**Analgesic consumption in the PACU**							
*WHO1*	n (%)	4 (5.63)	1 (4.17)		0 (0.00)	3 (13.04)	
*WHO3*	n (%)	29 (40.85)	8 (33.33)	0.38	12 (50.00)	9 (39.13)	0.561
*Total patients*	n (%)	33 (46.48)	9 (37.50)	0.561	12 (50.00)	12 (52.17)	0.772
**Analgesic consumption after discharge from PACU**							
*WHO1*	n (%)	40 (56.34)	13 (54.17)		15 (62.50)	12 (52.17)	
*WHO3*	n (%)	4 (5.63)	1 (4.17)	1.000	2 (8.33)	1 (4.35)	1.000
*Total patients*	n (%)	44 (61.97)	14 (58.33)	0.547	17 (70.83)	13 (56.52)	0.371
**Cumulative analgesic consumption**							
*WHO1*	n (%)	23 (32.39)	8 (33.33)		6 (25.00)	9 (39.13)	
*WHO3*	n (%)	30 (42.25)	9 (37.50)	0.561	12 (50.00)	9 (39.13)	0.561
*Total patients*	n (%)	53 (74.65)	17 (70.83)	1.000	18 (75.00)	18 (78.26)	1.000
**Number of patients with adverse events in the PACU** ***n (%)***							
*Nausea*	n (%)	5 (7.0)	2 (8.3)	1.000	2 (8.3)	1 (4.3)	1.000
*Vomiting*	n (%)	2 (2.8)	1 (4.2)	1.000	1 (4.2)	0 (0.0)	1.000
*Shivering*	n (%)	8 (11.3)	3 (12.5)	1.000	2 (8.3)	3 (13.0)	0.666
*Bleeding*	n (%)	1 (1.4)	0 (0.0)	—	0 (0.0)	1 (4.3)	0.489
*Vertigo*	n (%)	3 (4.2)	2 (8.3)	1.000	1 (4.2)	0 (0.0)	1.000
*Chills*	n (%)	1 (1.4)	0 (0.0)	—	0 (0.0)	1 (4.3)	0.489
*Crying*	n (%)	2 (2.8)	0 (0.0)	0.489	2 (8.3)	0 (0.0)	0.489
*Anxiety*	n (%)	3 (4.2)	2 (8.3)	0.489	0 (0.0)	1 (4.3)	0.489
*Fear*	n (%)	1 (1.4)	1 (4.2)	1.000	0 (0.0)	0 (0.0)	—
*Tiredness*	n (%)	1 (1.4)	0 (0.0)	1.000	1 (4.2)	0 (0.0)	1.000
*Total number of patients with AEs in the PACU*	n (%)	21 (29.6)	9 (37.5)	0.534	6 (25.0)	6 (26.1)	1.000
**Total number patients with adverse events** ***n (%)***							
*Nausea*	n (%)	25 (35.2)	8 (33.3)	0.517	11 (45.8)	6 (26.1)	0.111
*Vomiting*	n (%)	11 (15.5)	3 (12.5)	0.139	7 (29.2)	1 (4.3)	**0.018** ^**#**^
*Shivering*	n (%)	13 (18.3	6 (25.0)	0.717	4 (16.7)	3 (13.0)	0.684
*Flatulences*	n (%)	19 (26.8)	6 (25.0)	1.000	5 (20.8)	8 (34.8)	0.489
*Pyrosis*	n (%)	2 (2.8)	0 (0.0)	0.457	1 (4.2)	1 (4.3)	1.000
*Abdominal cramps*	n (%)	3 (4.2)	2 (8.3)	0.489	0 (0.0)	1 (4.3)	1.000
*Bleeding*	n (%)	4 (5.6)	0 (0.0)	0.457	1 (4.2)	3 (13.0)	0.604
*Vertigo*	n (%)	5 (7.0)	2 (8.3)	1.000	2 (8.3)	1 (4.3)	0.603
*Headache*	n (%)	3 (4.2)	1 (4.2)	0.593	2 (8.3)	0 (0.0)	0.229
*Hypertension*	n (%)	2 (2.8)	1 (4.2)	1.000	0 (0.0)	1 (4.3)	1.000
*Total number of patients with AEs*	n (%)	47 (66.2)	16 (66.7)	1.000	17 (70.8)	14 (17.4)	0.451

APU: press plaster acupressure, CON: control, ACU: press needle acupuncture, n: case number, M: median, IQR: interquartile range, STAI: State-Trait Anxiety Inventory, min^−1^: per minute, mmHg: millimeters of mercury, BIS: bispectral index, min: minute(s), Conc.: concentration, TCIoff: stop of target controlled infusion, WHO1 non-opioid analgesics according to the world health organization e.g. metamizol, WHO3 opioid analgesics i.e. piritramid, *significant on an alpha level of 5% according to Kruskal-Wallis-Test, ^#^significantly different from the CON group on an alpha level of 5%, post-hoc pairwise group comparisons were performed by Dunn’s Test.

**Figure 3:**
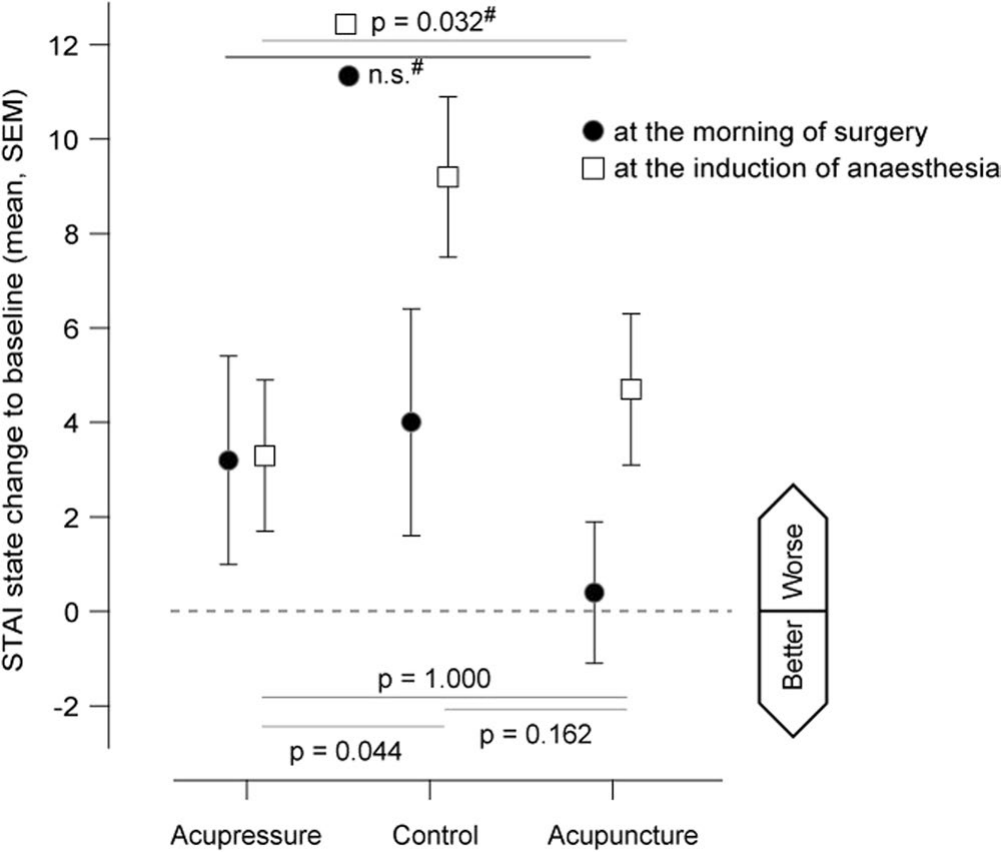
Change in State Anxiety to baseline. The figure displays mean changes with standard error of the mean (SEM) in state anxiety (in scoring points) from baseline in the three study groups (APU: press plaster acupressure, CON: control, ACU: press needle acupuncture). Positive changes indicate an increase in state anxiety. Filled circles indicate changes in anxiety at the morning of the day of surgery. Open squares indicate changes in anxiety prior to the induction anaesthesia, i.e. placement of the oxygen mask. P-Values are given for the comparison between all three groups according to analysis of variance (ANOVA; marked with #) and post-hoc pairwise comparisons at the induction of anaesthesia as performed by unpaired t-Test.

Regarding pre-operative anxiety, there were no differences in state and trait anxiety between groups at baseline, nor at admission in the morning of the day of surgery or directly before induction (see Table 4). Repeated measures ANOVA of state anxiety (time × group; 3 × 3) revealed a trend towards an interaction between time and intervention group (p = 0.067). Changes in state anxiety from baseline at the time point of induction showed clinically remarkable differences when comparing the CON (mean (SD) 9 (8)) to the ACU (5 (7)), or the APU group (3 (8); Fig. 3).

The BIS index indicated significant differences between groups with regard to sedation at induction of anaesthesia (p = 0.029), and when turning off the TCI (p = 0.020). Post-hoc tests revealed, that at induction of anaesthesia, patients in the ACU group had lower BIS when compared to the CON group (median (IQR) 97 (96–98) vs 98 (98–98); p = 0.008). When turning off the TCI, patients in both the ACU (38 (31–50); p = 0.015) and the APU group (42 (27–50); p = 0.016) showed significantly higher BIS scores when compared to the CON group (32 (24–39); Table 4).

In addition, there was a significant shorter time between the stop of the TCI and extubation in the ACU (median (IQR) 12 (7–22) min; p = 0.028) and the APU group (13 (9–20) min; p = 0.017) when compared to the CON group (19 (14–26) min; Kruskal-Wallis-Test p = 0.029; Table 3).

No differences of anaesthetic drugs or depth of anaesthesia following 5 minutes after induction between groups could be observed (Table 4). The median total amount of sufentanyl was 44 (IQR: 40–49) µg and of propofol 1197 (IQR: 975–1594) mg with no differences between groups. However, duration of surgery differed significantly between groups (p = 0.035 Kruskal-Wallis-Test). Pairwise group comparisons revealed significantly shorter surgery time in the CON group (median (IQR) 56 (44–71)) than in the APU (73 (55–115); p = 0.028) and ACU group (85 (52–107); p = 0.023). Time from induction to turning off the TCI was also different between groups (p = 0.048; Kruskal-Wallis-Test) with significantly shorter times in the CON group (92 (84–103) than in the APU group (105 (95–159); p = 0.015).

**Figure 4:**
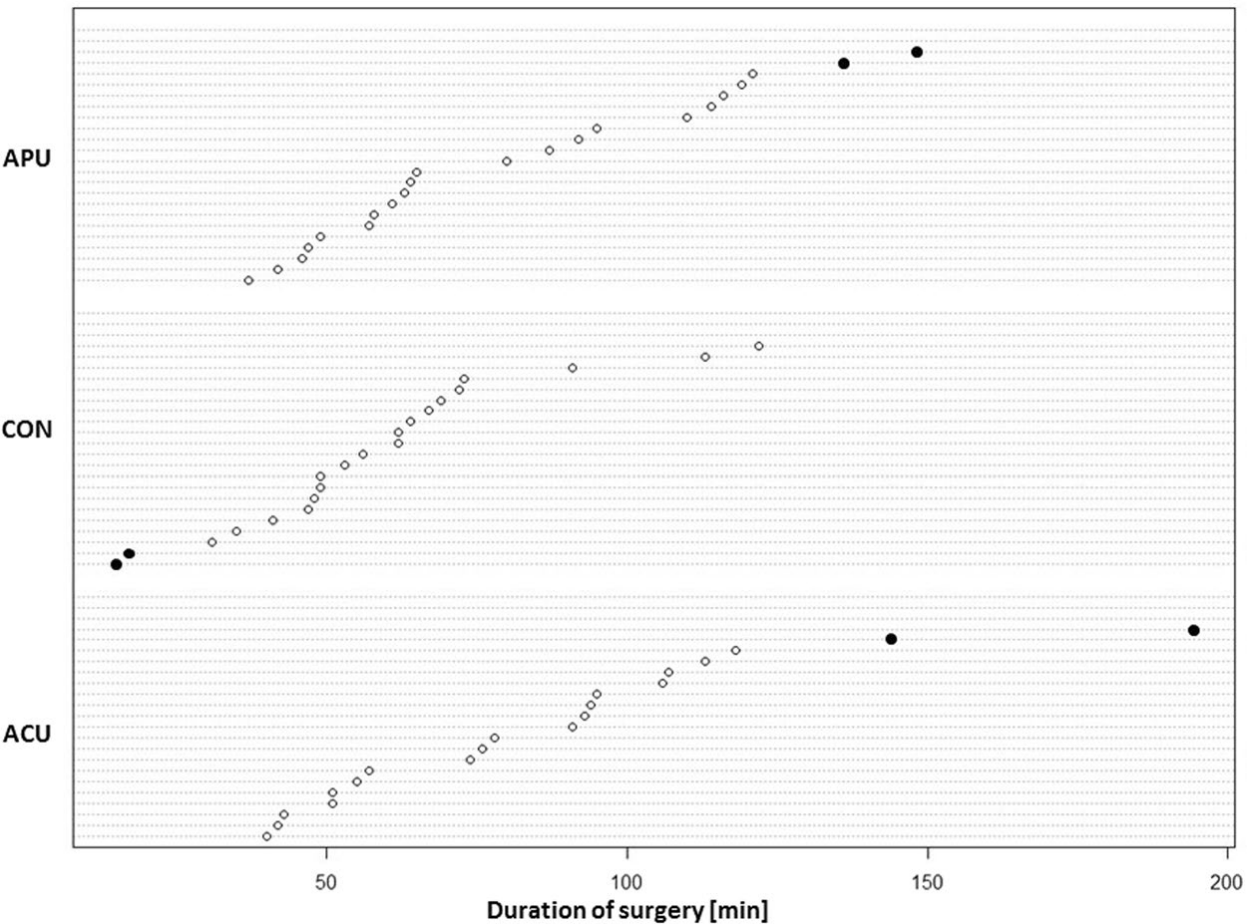
Surgery times. Extreme values were identified from the graphical display of individual surgery times per group.

To address possible bias introduced by unequal surgery durations, a sensitivity analysis excluding cases with extremely short, extremely long or missing surgery times was performed (Fig. 4). The overall group difference in time from extubation to ‘ready for discharge’ remained significant (Kruskal-Wallis-Test p = 0.015). Median time in the ACU group remained significantly shorter (13.5 min) than in the control group (p = 0.004), but the time difference to the APU group did not meet statistical significance (p = 0.108). The overall group comparison in time to extubation remained significant (p = 0.037), and pairwise comparisons showed a shorter times in the ACU (p = 0.025) and APU group (p = 0.027) when compared to CON group.

There were no group differences in vital signs at any time and in pain intensity during the whole post-operative period (Table 4 and Supplementary Table 1). Groups neither differed in regard to total analgesic consumption (in total 52 consumptions; Table 4).

There was a lower number of patients suffering from PONV in the APU and the ACU group than in the CON group (Table 4). Eleven patients in the CON group suffered from nausea and seven of them also from vomiting, whereas eight patients in the APU group and six patients in the ACU group, suffered from nausea of which three and one also suffered from vomiting, respectively. The incidence of vomiting was significantly reduced in the ACU when compared to the CON group (Fisher-Test p = 0.018).

There were no severe side effects caused by one of the study interventions. One patient in the ACU group experienced a minor adverse event in terms of slight redness at the needling site (GV 26).

## Discussion

The results of the AcuARP trial show that acupuncture applied by press needles can be effective in improving patients’ post-anaesthetic recovery. Median time from extubation to ‘ready for discharge’ from the PACU was 16 min shorter in ACU group than in the control group. In contrast, acupressure did not substantially affect the time from extubation to ‘ready for discharge’. This suggests specific effects elicited by the needle stimulus which render acupuncture superior over acupressure. This may be related to the overall stronger sensory input elicited by the penetrating needling stimulus when compared to non-penetrating pressure alone. Penetrating needles mechanically stimulate cells followed by a release of endogenous substances, that have been shown to activate neural afferents (A and C fibres)^19^, a way of action which may not be achieved by simple non-penetrating pressure.

To our knowledge, this is the first study evaluating the time between extubation and discharge from the PACU by means of standardised validated questionnaires^11^. There are hints from other studies using different outcome parameter for evaluating post anaesthetic recovery. Meta-analytic evidence from 7 studies with 540 patients indicates that acupuncture is efficient and safe for improving recovery after colorectal cancer surgery, namely time to first flatus and time to first defecation^20^. Additionally, in patients undergoing ophthalmic surgeries, acupuncture was shown to reduce time to spontaneous eye opening, time to extubation, and time to follow commands when compared to sham or control^21^. To prevent adverse events from electrical currents, the use of bipolar laparoscopic systems is preferable. When choosing monopolar systems, one should take a sufficient distance to the needles into account, and the current should be derived in the opposite direction. In this study no burns or electricity related adverse events could be observed.

Further observations in our study are also indicative for the beneficial effects of acupuncture during the post-operative recovery period. Stimulation at the acupuncture point GV26 seems particularly effective in promoting awakening independent of needle specific effects. Both, patients in the ACU and the APU group, could be extubated 6–7 minutes earlier after turning off the TCI-pump than patients in the CON group. This observation is in line with results from the trial in patients undergoing ophthalmic surgery mentioned above, as well as with animal experiments in snapping turtles^15^ and in rabbits^22^. Acupuncture is supposed to activate noradrenergic neurotransmission in the brain which in turn reduces the central nervous depressive activity of anaesthetics^22^. However, the faster awakening in the two intervention groups could also be due to the fact that the anaesthetic state was already less deep at the time-point when anaesthetic infusion was stopped; as indicated by the BIS.

The increase of state anxiety during the period from admission to induction of anaesthesia was less pronounced in patients receiving acupuncture or acupressure than in patients in the control group (see Fig. 3). BIS scores indicated a trend towards an accelerated sedation and an earlier regain of consciousness when receiving acupuncture or acupressure. The observed effects on anxiety are in line with previously reported effects of ear acupuncture on anxiety^23^. A recent study showed the individualization of complementary treatments to be an important factor boosting the effect on pre-operative anxiety^24^. It might thus be possible -as our patients indicated overall low levels in state and trait anxiety- that the general setting, and being taken care of by the study team, helped minimising anxiety.

Furthermore, there was a trend towards lower rates of nausea and vomiting (ACU n = 6, CON n = 11) as well as less demand for strong analgesics (WHO III, ACU n = 9, CON n = 12). However, this study was not designed to evaluate the effect of acupuncture on PONV. The strong floor effect, the high rate of patients not experiencing PONV, might have masked the effect of acupuncture on PONV and post-operative analgesic consumption whose evidence is well-established^9^.

A particular strength of our trial is that the impact of differences in anaesthetic consumption can be ruled out. The use of TCI pumps allowed to objectively monitor the amount of applied drugs. At the time when the TCI pump was turned off, there were neither differences in regard to the estimated effect-site concentrations nor the estimated time to awakening (Table 4). In order to minimize bias from individually administered anaesthetic drugs, effect-site concentrations of anaesthetics and opioids in the TCI were set equally for all patients^25^. Effect-site concentrations chosen in our study were already at the lower margin of the clinically used range. Thus, an estimation of an intraoperative anaesthetic sparing effect through one of the interventions was not possible. Studies investigating the effects of acupuncture on anaesthetic consumption mainly focus on either volatile anaesthetics^4^ or opioids^5^ as a primary outcome.

The observed number of acupuncture-related side effects -one single event of redness at the acupuncture site- is in accordance with the assumption that acupuncture is a safe intervention^26^.

As variations in the effect size of acupuncture in different trials are driven predominantly by differences in treatments received by the control group rather than by differences in the characteristics of acupuncture treatment^27^, the number of postoperative visits, time spent with the patients, and attention was similarly offered in all groups. Thus, bias introduced by differences in patient care between study groups is unlikely and group differences observed seem rather linked to physiological mechanisms elicited by the peripheral stimulation in the intervention groups. The trend towards shorter times to extubation may be mediated by palpation upon the point GV26, as stimulated by the anesthetists’ and their anesthetic teams during the awakening phase, whereas shortening the recovery time seemed to be a needling-specific effect.

## Limitations

Limitations include the restricted sample size and limited external validity. This study was designed to detect changes in the post-operative recovery, i.e. the period between extubation and discharge from PACU, where patients are supposed to be most vulnerable. Conclusions on secondary outcomes need to be taken carefully.

Despite the use of a computer-based randomisation procedure surgery times were different between study groups. Although TCI-calculated times to awakening suggest comparability between groups, we cannot rule out, that the differences in the duration of surgery may have influenced study results. However, as indicated by the sensitivity analyses varying surgery times seem to have only marginally influenced identified group differences in recovery times.

Despite the convincing evidence that press-needles and press-plasters can neither be distinguished by patients nor by assessors^12^, it is not certain whether blinding was maintained throughout the study period as blinding credibility was not assessed in particular. Further bias may have resulted from the fact that the CON group could be visibly identified by assessors. This is known shortcoming studies addressing the add-on effect of acupuncture, and we are aware that placebo controls in acupuncture studies are of limited knowledge gain^28^. Nevertheless, the application of press-plasters as a control procedure is in line with recent suggestions, avoiding needling of the skin at non acupuncture points^29^, and the comparison of the ACU and APU to the CON group was necessary to quantify the effects of penetrating and non-penetrating acupuncture point stimulation.

As reported previously^11^, the point selection included points that had been described to reduce anxiety, promote awakening, and reduce pain and anaesthesia-related symptoms such as PONV. The decision was made on the basis of existing evidence (e.g. PC6 and PONV)^9^, expert opinion, and tradition. The use of press needles turned out to be feasible even in the peri-operative setting, and the chosen points seemed appropriate to address proposed indications. However, it is debateable, if acupuncture applied with common acupuncture needles resulting in higher stimulation intensity could cause larger treatment effects. The impact of needling depth, needle diameter and stimulation intensity on acupuncture effects are still under debate, but expert consensus suggests that larger treatment effects are achieved by deep and strong needling stimuli^30^. In addition, other point regimens might be equally effective^31^.

Further, our results are limited to a group of women receiving laparoscopic surgery, and can therefore not simply be extrapolated to other patient groups. However, we show the possibility to implement well-designed acupuncture research in a standard setting and encourage to do so for other indications as well.

## Conclusions

This study shows for the first time that acupuncture can shorten the time from extubation to ‘ready for discharge’ from the PACU in gynaecological laparoscopy. Further effects of acupuncture on secondary outcomes of recovery, i.e. shorter time to extubation (awakening) and reduced incidence of vomiting, need to be carefully interpreted. As recovery is a core process goal of surgery, multidisciplinary evidence-based programs can be encouraged to consider acupuncture as an element in gynaecological laparoscopy.

## Electronic supplementary material

Supplementary Table 1
