# Supplementary material for: Acupuncture reduces the time from extubation to ‘ready for discharge’ from the post anaesthesia care unit: results from the randomised controlled AcuARP trial
Source: Sci Rep. 2018 Oct 24;8:15734. doi: 10.1038/s41598-018-33459-y (PMC6200780; doi:10.1038/s41598-018-33459-y)
Supplement: Supplementary file 1 — Supplementary Table 1 [file 41598_2018_33459_MOESM1_ESM.docx]

Supplementary Table 1: Vital signs

|  | | **Total (n = 71)** | **APU (n = 24)** | | | **CON (n = 24)** | | | **ACU (n = 23)** | | | **p-value** | |
| --- | --- | --- | --- | --- | --- | --- | --- | --- | --- | --- | --- | --- | --- |
| **Vital signs at 1^st^ visit** | |  |  | | |  | | |  | | |  | |
| *Heart rate (min^-1^)* | *n* | 60 | 20 | | | 20 | | | 20 | | |  | |
|  | *M (IQR)* | 76.00 (66.00 - 80.75) | 72.50 (64.25 - 80.75) | | | 78.50 (68.50 - 81.00) | | | 77.00 (70.75 - 80.00) | | | 0.704 | |
| *Systolic blood pressure (mmHg)* | *n* | 59 | 19 | | | 20 | | | 20 | | |  | |
|  | *M (IQR)* | 122.0 (111.0 - 134.0) | 125.0 (111.0 - 140.0) | | | 121.5 (110.0 - 132.8) | | | 121.0 (112.8 - 136.3) | | | 0.834 | |
| *Diastolic blood pressure (mmHg)* | *n* | 59 | 19 | | | 20 | | | 20 | | |  | |
|  | *M (IQR)* | 80.0 (70.0 - 86.0) | 80.0 (72.0 - 89.0) | | | 79.5 (70.0 - 85.5) | | | 80.0 (70.0 - 85.3) | | | 0.909 | |
| **Vital signs at the time of extubation** | | |  | | |  | | |  | | |  | |
| *Heart rate (min^-1^) at extubation* | *n* | 71 | 24 | | | 24 | | | 23 | | |  | |
|  | *M (IQR)* | 78.0 (70.0 - 89.0) | 75.5 (68.5 - 86.5) | | | 80.0 (70.8 - 91.0) | | | 78.0 (67.0 - 87.0) | | | 0.526 | |
| *Systolic blood pressure at extubation (mmHg)* | *n* | 71 | 24 | | | 24 | | | 23 | | |  | |
|  | *M (IQR)* | 115.0 (106.0 - 129.0) | 124.5 (109.8 - 142.3) | | | 112.5 (103.3 - 125.5) | | | 112.0 (103.0 - 120.0) | | | 0.090 | |
| *Diastolic blood pressure at extubation (mmHg)* | *n* | 71 | 24 | | | 24 | | | 23 | | |  | |
|  | *M (IQR)* | 68.0 (60.0 - 78.0) | 76.0 (66.0 - 80.8) | | | 69.0 (58.5 - 76.0) | | | 64.0 (56.0 - 73.0) | | | 0.234 | |
| **Vital signs at the arrival at PACU**  **Vital signs at the arrival at PACU** | |  |  | | |  | | |  | | |  | |
| *Heart rate (min^-1^) at extubation* | *n* | 71 | 24 | | | 24 | | | 23 | | |  | |
|  | *M (IQR)* | 79.0 (71.0 - 88.0) | 75.0 (71.0 - 83.5) | | | 81.0 (73.3 - 92.3) | | | 82.0 (71.0 - 100.0) | | | 0.132 | |
| *Systolic blood pressure at extubation (mmHg)* | *n* | 71 | 24 | | | 24 | | | 23 | | |  | |
|  | *M (IQR)* | 114.0 (107.0 - 125.0) | 116.5 (106.0 - 126.8) | | | 119.5 (110.3 - 129.3) | | | 110.0 (105.0 - 117.0) | | | 0.097 | |
| *Diastolic blood pressure at extubation (mmHg)* | *n* | 71 | 24 | | | 24 | | | 23 | | |  | |
|  | *M (IQR)* | 67.0 (61.0 - 78.0) | 69.0 (58.5 - 80.5) | | | 67.0 (62.0 - 81.0) | | | 65.0 (61.0 - 74.0) | | | 0.579 | |
| *Saturation (% O_2_)extubation* | *n* | 71 | 24 | | | 24 | | | 23 | | |  | |
|  | *M (IQR)* | 97.0 (95.0 - 98.0) | 96.5 (95.0 - 98.0) | | | 96.0 (94.0 - 98.0) | | | 97.0 (94.0 - 99.0) | | | 0.588 | |
| **Vital signs at the discharge from PACU**  **Vital signs at the arrival at PACU** | | |  | | |  | | |  | | |  | |
| *Heart rate (min^-1^) at extubation* | *n* | 71 | 24 | | | 23 | | | 23 | | |  | |
|  | *M (IQR)* | 69.5 (63.0 - 80.5) | 66.5 (59.3 - 77.3) | | | 69.0 (63.0 - 79.0) | | | 72.0 (66.0 - 84.0) | | | 0.200 | |
| *Systolic blood pressure at extubation (mmHg)* | *n* | 71 | 24 | | | 23 | | | 23 | | |  | |
|  | *M (IQR)* | 116.0 (106.8 - 126.3) | 120.0 (111.8 - 138.0) | | | 113.0 (105.0 - 124.0) | | | 112.0 (105.0 - 120.0) | | | 0.075 | |
| *Diastolic blood pressure at extubation (mmHg)* | *n* | 71 | 24 | | | 23 | | | 23 | | |  | |
|  | *M (IQR)* | 68.5 (62.8 - 77.3) | 70.0 (65.3 - 78.0) | | | 67.0 (64.0 - 78.0) | | | 64.0 (60.0 - 74.0) | | | 0.201 | |
| **Post-operative vital signs** | |  | | |  | | |  | | |  | |  |
| *Heart rate (min-1) 6 h post-surgery* | n | 70 | | 24 | | | 24 | | | 22 | | |  |
|  | *M (IQR)* | 78.0 (70.8 - 84.3) | | 75.5 (69.3 - 87.5) | | | 80.0 (71.3 - 80.8) | | | 79.5 (70.3 - 86.3) | | | 0.767 |
| Systolic blood pressure (mmHg) 6 h post-surgery | n | 71 | | 24 | | | 24 | | | 23 | | |  |
|  | *M (IQR)* | 111.0 (105.0 - 124.0) | | 115.0 (108.0 - 129.5) | | | 109.0 (100.0 - 122.8) | | | 116.0 (100.0 - 123.0) | | | 0.235 |
|  |  |  | |  | | |  | | |  | | |  |
| *Diastolic blood pressure (mmHg) 6 h post-surgery* | n | 71 | | 24 | | | 24 | | | 23 | | |  |
|  | *M (IQR)* | 69.0 (61.0 - 80.0) | | 70.0 (65.0 - 80.0) | | | 68.0 (60.0 - 70.0) | | | 70.0 (57.0 - 80.0) | | | 0.290 |
|  |  |  | |  | | |  | | |  | | |  |
| *Heart rate (min-1)*  *d1 8°°* | n | 67 | | 22 | | | 23 | | | 22 | | |  |
|  | *M (IQR)* | 76.0 (68.0 - 84.0) | | 77.0 (69.5 - 86.0) | | | 72.0 (68.0 - 80.0) | | | 79.5 (65.5 - 88.5) | | | 0.213 |
|  |  |  | |  | | |  | | |  | | |  |
| Systolic blood pressure (mmHg) d1 8°° | n | 68 | | 22 | | | 23 | | | 23 | | |  |
|  | *M (IQR)* | 106.5 (101.0 - 120.0) | | 106.0 (104.8 - 123.0) | | | 109.0 (101.0 - 122.0) | | | 104.0 (98.0 - 114.0) | | | 0.521 |
| *Diastolic blood pressure (mmHg) d1 8°°* | n | 68 | | 22 | | | 23 | | | 23 | | |  |
|  | *M (IQR)* | 63.5 (57.3 - 70.0) | | 64.0 (57.0 - 71.3) | | | 67.0 (60.0 - 70.0) | | | 62.0 (55.0 - 70.0) | | | 0.739 |
| *Heart rate (min-1)*  *d1 20°°* | n | 65 | | 24 | | | 21 | | | 20 | | |  |
|  | *M (IQR)* | 78.0 (70.0 - 85.0) | | 78.5 (72.0 - 84.0) | | | 74.0 (65.0 - 82.5) | | | 84.0 (69.3 - 88.0) | | | 0.095 |
| Systolic blood pressure (mmHg) d1 20°° | n | 66 | | 24 | | | 21 | | | 21 | | |  |
|  | *M (IQR)* | 117.0 (106.0 - 130.0) | | 122.0 (108.5 - 137.8) | | | 115.0 (110.0 - 127.0) | | | 110.0 (99.5 - 121.0) | | | 0.127 |
| *Diastolic blood pressure (mmHg) d1 20°°* | n | 66 | | 24 | | | 21 | | | 21 | | |  |
|  | *M (IQR)* | 70.0 (60.0 - 77.3) | | 73.5 (60.3 - 80.0) | | | 70.0 (60.0 - 75.0) | | | 68.0 (57.0 - 78.5) | | | 0.348 |
| *Heart rate (min-1)*  *d2 8°°* | n | 66 | | 23 | | | 22 | | | 21 | | |  |
|  | *M (IQR)* | 75.0 (68.0 - 81.0) | | 75.0 (70.0 - 82.0) | | | 74.5 (66.0 - 80.3) | | | 76.0 (64.5 - 82.5) | | | 0.866 |
| Systolic blood pressure (mmHg) d2 8°° | n | 66 | | 23 | | | 21 | | | 22 | | |  |
|  | *M (IQR)* | 116.0 (106.5 - 128.0) | | 116.0 (110.0 - 127.0) | | | 116.0 (110.0 - 127.5) | | | 116.5 (101.5 - 128.5) | | | 0.935 |
|  |  |  | |  | | |  | | |  | | |  |
| *Diastolic blood pressure (mmHg) d2 8°°* | n | 66 | | 23 | | | 21 | | | 22 | | |  |
|  | *M (IQR)* | 68.5 (62.0 - 73.0) | | 68.0 (60.0 - 72.0) | | | 70.0 (62.0 - 73.0) | | | 69.5 (62.0 - 73.3) | | | 0.879 |
| *Heart rate (min-1)*  *d2 20°°* | n | 49 | | 18 | | | 15 | | | 16 | | |  |
|  | *M (IQR)* | 73.0 (67.0 - 80.5) | | 72.0 (66.5 - 80.0) | | | 73.0 (68.0 - 77.0) | | | 78.5 (64.8 - 84.3) | | | 0.691 |
| Systolic blood pressure (mmHg) d2 20°° | n | 50 | | 19 | | | 15 | | | 16 | | |  |
|  | *M (IQR)* | 119.0 (111.0 - 130.0) | | 118.0 (112.0 - 130.0) | | | 120.0 (108.0 - 130.0) | | | 119.0 (111.8 - 130.0) | | | 0.977 |
| *Diastolic blood pressure (mmHg) d2 20°°* | n | 50 | | 19 | | | 15 | | | 16 | | |  |
|  | *M (IQR)* | 70.5 (65.0 - 80.0) | | 71.0 (62.0 - 78.0) | | | 70.0 (65.0 - 82.0) | | | 73.5 (68.0 - 80.8) | | | 0.627 |

APU: press plaster acupressure, CON: control, ACU: press needle acupuncture, n: case number, M: median, IQR: interquartile range, *min^-1^*: per minute, mmHg: millimeters of mercury, BIS: bispectral index, min: minute(s), Conc.: concentration, TCIoff: stop of target controlled infusion, * significant on an alpha level of 5% according to Kruskal-Wallis-Test, ^##^ significantly different from both other study groups on an alpha level of 5%, ^#^ significantly different from the CON group on an alpha level of 5%, post-hoc comparison performed by Mann-Whitney-U-Test
